# Valproic Acid-Induced Pancreatitis in a Pediatric Patient With 17q12 Duplication Syndrome

**DOI:** 10.7759/cureus.101694

**Published:** 2026-01-16

**Authors:** Yehya M Maitah, Alyssa J Snyder, Ian Maguire, Lyman Wu, Meghan Aabo

**Affiliations:** 1 Department of Pediatrics, Albany Medical College, Albany, USA; 2 Department of Pediatrics, Albany Medical Center, Albany, USA

**Keywords:** 17q12 duplication, acute pancreatitis (ap), depakote, drug-induced acute pancreatitis, drug reaction, pediatric pancreatitis, pediatric seizure

## Abstract

Drug-induced acute pancreatitis is an uncommon cause of acute pancreatitis. Among the common culprits is valproic acid (VPA) and its derivatives, i.e., Depakote. These medications are commonly prescribed for epilepsy, bipolar disorder, and migraines. Although VPA-induced pancreatitis is rare, it has a variable disease course and may progress to severe disease, which can become fatal. Our case reports a 14-year-old male with a history of 17q.12 duplication, focal epilepsy, autism spectrum disorder (ASD), and attention-deficit hyperactivity disorder (ADHD), who presented to the emergency department with acute abdominal pain, nausea, and decreased oral intake. Labs revealed elevated lipase (1,572 U/L) and amylase (137 U/L), and triglycerides and calcium levels were within the reference range. An abdominal ultrasound was negative. The patient was being treated for focal epilepsy with VPA, which had been increased from 500 mg twice daily to 500 mg in the morning and 625 mg in the evening one month prior. Other causes of pancreatitis were ruled out. Discontinuation of the VPA led to rapid clinical improvement and normalization of lab values. The Naranjo Adverse Drug Reaction Probability Scale indicated that this event was a “probable” adverse drug reaction. This adverse effect occurred with VPA monotherapy at therapeutic dosing in the absence of risk factors, in the setting of 17q12 duplication syndrome, ADHD, and ASD. The clear sequence of events between the increase in dosage, onset of symptoms, medication discontinuation, and resolution of symptoms strengthens the causal link between the drug and the reaction. VPA-induced pancreatitis, despite its low incidence, carries a high risk of severe disease and mortality. It is critical that clinicians monitor for this complication in patients taking VPA and its derivatives. This case expands the spectrum of patients who may be vulnerable to this adverse reaction and highlights the importance of swift identification and discontinuation of the drug.

## Introduction

Acute pancreatitis is one of the most common gastrointestinal conditions requiring hospitalization in the United States [1]. The disease course is variable, with 20-30% of people progressing to a severe form of the disease. Many causes of pancreatitis have been identified, including drugs [2]. Drugs cause between 0.1% and 0.2% of acute pancreatitis cases, with hundreds shown to cause this adverse reaction [3]. Valproic acid (VPA)-induced pancreatitis is a rare, potentially life-threatening cause of pancreatitis with a poorly understood etiology.

The incidence of this adverse effect has been estimated to be 1:40,000 according to clinical trials and post-marketing data [4]. Cases have been reported in both children and adults being treated for epilepsy, as well as bipolar disorder and migraines. Children seem to be disproportionately affected, with two-thirds of reported cases being children under the age of 18 years in a recent systematic review [5]. Median time to onset of VPA-induced pancreatitis varies, with cases occurring between days to years after beginning therapy [5].

Despite there being no apparent risk factors increasing the risk of developing pancreatitis secondary to VPA therapy, there has been an association with anti-epileptic polypharmacy [6]. Mortality is a significant risk in VPA-induced pancreatitis, with fatality rates potentially as high as 16% [5]. The U.S. Food and Drug Administration warns patients about pancreatitis as an adverse effect of VPA use in both children and adults, emphasizing that it may occur at any point during treatment and may be rapidly fatal [4]. Although VPA and its derivatives (Depakote) are widely used for the treatment of epilepsy, relatively few cases have been documented with acute pancreatitis as an adverse effect.

Most cases that have been reported involve patients on a multi-medication regimen for epilepsy and/or predisposing conditions. Our case describes a patient who was on VPA monotherapy with standard therapeutic dosing, no predisposing risk factors, and a 17q12 duplication syndrome. This genetic background has not been previously reported in the literature in association with VPA-induced pancreatitis.

## Case presentation

A 14-year-old male with a complex medical history, including 17q.12 duplication, focal epilepsy, autism spectrum disorder (ASD), and attention-deficit hyperactivity disorder (ADHD), presented to the emergency department with an acute onset of severe abdominal pain. The patient developed severe upper abdominal and periumbilical pain three days prior to presentation, described as unlike any pain he had previously experienced. The pain was associated with nausea and decreased oral intake. Initial symptomatic treatment with calcium carbonate (Tums) provided no relief, prompting his mother to seek medical attention. The patient was initially evaluated at a local emergency department, where an abdominal ultrasound was unremarkable, but serum lipase was significantly elevated at 1,572 U/L. Given the concern for acute pancreatitis and the patient's complex neurological comorbidities, he was transferred to our tertiary pediatric center.

One month prior to the presentation, the patient had been evaluated by pediatric neurology for increased seizure frequency from one to two per month to more than two seizures per month. His VPA regimen was adjusted from 500 mg twice daily to 500 mg in the morning and 625 mg in the evening. Despite this increase, the patient experienced five seizures over the subsequent 21 days, leading to consideration of adding zonisamide for breakthrough seizures.

On presentation to our emergency department, the patient appeared uncomfortable with achy, non-radiating midabdominal pain and right lower quadrant pain that worsened with ambulation. Physical examination was notable for the absence of costovertebral angle tenderness, guarding, or rebound tenderness. The patient denied fever, chills, vomiting, diarrhea, constipation, trauma, or lethargy.

Initial laboratory evaluation at the outside hospital revealed a markedly elevated lipase level of 1,572 U/L (normal: 10-140 U/L), alkaline phosphatase of 424 U/L, aspartate aminotransferase (AST)/alanine aminotransferase (ALT) of 46/17 U/L, and total bilirubin level of 1.3 mg/dL (Table 1). Complete blood count and basic metabolic panel were within normal limits. Triglycerides were normal at 34 mg/dL. CT of the abdomen and pelvis, as well as abdominal ultrasound, were unremarkable. Repeat laboratory values at our institution showed persistent elevation of pancreatic enzymes with amylase at 137 U/L and lipase at 190 U/L, along with mildly elevated AST at 33 U/L and stable total bilirubin at 1.2 mg/dL. The respiratory viral panel was negative.

**Table 1:** Initial diagnostic laboratory workup for suspected acute pancreatitis. Values marked with H or L indicate results above or below the reference range, respectively. All laboratory values were obtained at the time of presentation. Reference ranges reflect institutional standards. Hgb: hemoglobin; Hct: hematocrit; MCV: mean corpuscular volume; MCH: mean corpuscular hemoglobin; MCHC: mean corpuscular hemoglobin concentration; RDW-CV: red cell distribution width - coefficient of variation; Plt: platelets; MPV: mean platelet volume; BUN: blood urea nitrogen; AST: aspartate aminotransferase; ALT: alanine aminotransferase.

Complete blood count				
Component	Value	Reference range	Units	Flag
WBC	6.37	3.13 - 12.43	K/cmm	
RBC	4.12	3.97 - 5.88	M/cmm	
Hgb	13.5	11.2 - 16.4	g/dL	
Hct	39.8	33.7 - 48.7	%	
MCV	97	74 - 94	fl	H
MCH	32.8	23.1 - 31.7	pg	H
MCHC	33.9	30.6 - 35.3	g/dL	
RDW-CV	13.9	11.5 - 16.5	%	
Plt	199	152 - 426	K/cmm	
MPV	9.7	8.9 - 12.8	fl	
Comprehensive metabolic panel				
Component	Value	Reference range	Units	Flag
Sodium	137	136 - 145	mmol/L	
Potassium	4.1	3.5 - 5.0	mmol/L	
Chloride	101	96 - 110	mmol/L	
CO2	26	22 - 32	mmol/L	
Glucose	87	70 - 99	mg/dL	
BUN	17	7.0 - 21	mg/dL	
Creatinine	0.75	0.42 - 0.76	mg/dL	
Total protein	7	6.6 - 8.3	mg/dL	
Albumin	4.6	4.2 - 5.0	g/dL	
Alkaline phosphatase	424	119 - 440	U/L	
AST	46	24 - 49	U/L	
ALT	17	22 - 49	U/L	L
Bilirubin	1.3	0.8	mg/dL	H
Calcium	10.1	8.9 - 10.2	mg/dL	
Other labs				
Component	Value	Reference range	Units	Flag
Lipase	1,572	148	U/L	H
Amylase	137	23 - 85	U/L	
Triglycerides	34	<90	mg/dL	

The patient was admitted with a diagnosis of acute pancreatitis, likely secondary to VPA therapy. VPA was discontinued, and the patient was transitioned to lacosamide for seizure control. Conservative management with bowel rest, intravenous fluids, and analgesics was initiated, and the patient's clinical picture improved. Laboratory monitoring showed improvement in pancreatic enzymes, with lipase decreasing to 98 U/L by discharge. No seizure activity was observed during the transition to lacosamide.

The patient was discharged home in stable condition with resolution of symptoms and normalized pancreatic enzymes. He was scheduled for follow-up with pediatric neurology in two to three months to assess seizure control on the new antiepileptic regimen (Table 2).

**Table 2:** Clinical timeline demonstrating the relationship between valproic acid (VPA) dose adjustment and onset of pancreatitis. BID: twice per day.

Day	Event
–30	Valproic acid dose increased from 500 mg BID → 500 mg AM/625 mg PM due to increased seizure frequency.
–21 to –7	Patient experiences 5 seizures over 21 days despite dose increase; consideration of adding zonisamide.
–3	Onset of new severe upper abdominal and periumbilical pain, associated with nausea and decreased oral intake.
0 (Initial ED visit)	Presents to outside hospital ED; lipase = 1,572 U/L; amylase elevated; abdominal ultrasound normal. Transferred to a tertiary pediatric center.
0–1	Repeat labs: amylase = 137 U/L, lipase = 190 U/L. Diagnosis of acute pancreatitis made.
1	VPA discontinued; lacosamide initiated for seizure control. Supportive care started (IV fluids, bowel rest, analgesics).
1–2	Clinical symptoms improve rapidly; abdominal pain decreases.
2–3	Pancreatic enzymes trend downward; lipase reaches 98 U/L. No further seizure activity observed.
3–4	Symptoms resolved; patient is tolerating oral intake.
4	Patient discharged home in stable condition with follow-up arranged with pediatric neurology.

## Discussion

VPA and its derivatives (i.e., Depakote) are medications used in the management of epilepsy, bipolar disorder, and migraine prophylaxis. Although they are generally well tolerated, several uncommon, yet life-threatening adverse effects have been identified [7]. Acute pancreatitis is a rare adverse effect of VPA that can be rapidly fatal without timely intervention. Fewer than 150 cases have been described in the literature, with around two-thirds of the cases occurring in the pediatric population. Cases such as this one highlight the importance of early identification and maintaining a high level of suspicion in the pediatric population.

Although the exact mechanism is not completely understood, current evidence supports a possible non-dose-dependent mechanism. It has been demonstrated that VPA and its derivatives inhibit histone deacetylases (HDACs), which are involved in pancreatic acinar cell differentiation after injury. This impaired redifferentiation leads to delayed pancreatic regeneration and increases the persistence of acinar to ductal metaplasia, predisposing to acute inflammation and longer recovery after injury [8]. Other possible mechanisms include direct damage to pancreatic acinar cells, mitochondrial dysfunction, and the generation of toxic metabolites; however, these mechanisms are less studied in the literature [6,9].

In this case, diagnosis was made as a combination of clinical judgement based on the temporal association between the increase in VPA dosage, as well as laboratory values and radiological studies indicating signs of acute pancreatitis. Common causes of acute pancreatitis were ruled out through history and laboratory values. The Naranjo Adverse Drug Reaction Probability Scale is a widely used causality assessment of adverse drug reactions [10,11]. Using the Naranjo Adverse Drug Reaction Probability Scale, the likelihood that this reaction was caused by VPA use is “probable” (Table 3). The recurrence risk of VPA-induced pancreatitis can be up to 80% [5]. Due to the high risk of recurrence of pancreatitis, it was highly recommended to discontinue the drug to prevent relapse of the patient's condition.

**Table 3:** Naranjo Adverse Drug Reaction Probability Scale applied to this case. Score: 9 or more = definite, 5-8 = probable, 1-4 = possible, and 0 or less = doubtful.

Question	Answer	Score
Are there previous conclusive reports of this reaction?	Yes	1
Did the adverse event appear after the suspected drug was administered?	Yes	2
Did the adverse reaction improve when the drug was discontinued, or a specific antagonist was given?	Yes	1
Did the adverse reaction reappear when the drug was readministered?	Not applicable	0
Are there alternative causes that could explain the reaction?	No	2
Did the reaction reappear when a placebo was given?	Not applicable	0
Was the drug detected in any body fluid in toxic concentrations?	Not measured	0
Was the reaction more severe when the dose was increased, or less severe when the dose was decreased?	Yes (after recent dose increase)	1
Did the patient have a similar reaction to the same or similar drugs in the past?	No	0
Was the adverse event confirmed by objective evidence (e.g., labs, imaging)?	Yes (labs, imaging)	1
Total score		8

This case is unique from previously described cases in many ways. Firstly, many previously published reports about patients experiencing VPA-induced pancreatitis involve multi-drug regimens for epilepsy. This patient was on VPA monotherapy at a standard therapeutic dosage. Additionally, he had no known risk factors, allergies, or hypersensitivities that could predispose him to acute pancreatitis. Presence of a 17q12 duplication syndrome in association with VPA-induced pancreatitis has not been previously described, possibly suggesting a previously unknown susceptibility, although more evidence is needed. In addition, this case features a patient who is diagnosed with ASD and ADHD, rarely described in the literature on previous cases, although the significance of these diagnoses is unclear. Furthermore, this case demonstrates a clear chronological timeline of events between the increase in VPA dosage and symptom onset, as well as rapid symptom resolution following discontinuation of the drug. These elements highlight the causal association between VPA and acute pancreatitis, while emphasizing the novelty of this case within the existing body of literature.

This report describes a single patient, which inherently limits generalizability. Despite evidence suggesting a temporal relationship between the increase in medication dosage, development of the condition, discontinuance of medications, and clinical resolution of symptoms, the lack of drug rechallenge prevents conclusive establishment of causation. Additionally, since serum valproate levels were not measured at the time of the patient’s initial presentation, the relationship between the drug level in circulation and pancreatic injury could not be evaluated. Absence of radiographic pancreatic abnormalities does not exclude early or mild pancreatitis and may limit characterization of disease severity.

## Conclusions

Acute pancreatitis is a rare, yet potentially life-threatening, reaction caused by the use of VPA and its derivatives. This case highlights the importance of monitoring for signs of pancreatitis with VPA use, irrespective of dose, treatment duration, and other known risk factors for acute pancreatitis. Pediatric populations seem to be especially at risk for this complication. This adverse effect is often fatal and has a high rate of recurrence. Because of this, immediate discontinuation is essential. The presence of a 17q12 duplication syndrome, in addition to diagnoses of ASD and ADHD, expands the spectrum of patients who may be at increased risk for this reaction. Continuing to be vigilant for cases like this will raise awareness for this complication, allowing for a more comprehensive understanding and more efficient monitoring.
